# Genomic variation in *Pseudomonas aeruginosa* clinical respiratory isolates with *de* *novo* resistance to a bacteriophage cocktail

**DOI:** 10.1128/spectrum.02149-24

**Published:** 2025-03-31

**Authors:** Stephanie A. Fong, George Bouras, Ghais Houtak, Roshan Nepal, Sholeh Feizi, Sandra Morales, Alkis J. Psaltis, Peter-John Wormald, Sarah Vreugde

**Affiliations:** 1 Department of Surgery - Otolaryngology Head and Neck Surgery, University of Adelaide, Adelaide, Australia; 2 AmpliPhi Australia, Brookvale, New South Wales, Australia; University of Pittsburgh School of Medicine, Pittsburgh, Pennsylvania, USA

**Keywords:** bacteriophage, *Pseudomonas aeruginosa*, cystic fibrosis, multidrug resistance

## Abstract

*Pseudomonas aeruginosa* is an opportunistic pathogen that can cause sinus infections and pneumonia in cystic fibrosis (CF) patients. Bacteriophage therapy is being investigated as a treatment for antibiotic-resistant *P. aeruginosa* infections. Although virulent bacteriophages have shown promise in treating *P. aeruginosa* infections, the development of bacteriophage-insensitive mutants (BIMs) in the presence of bacteriophages has been described. The aim of this study was to examine the genetic changes associated with the BIM phenotype. Biofilms of three genetically distinct *P. aeruginosa* strains, including PAO1 (ATCC 15692), and two clinical respiratory isolates (one CF and one non-CF) were grown for 7 days and treated with either a cocktail of four bacteriophages or a vehicle control for 7 consecutive days. BIMs isolated from the biofilms were detected by streak assays, and resistance to the phage cocktail was confirmed using spot test assays. Comparison of whole genome sequencing between the recovered BIMs and their respective vehicle control-treated phage-sensitive isolates revealed structural variants in two strains, and several small variants in all three strains. These variations involved a TonB-dependent outer membrane receptor in one strain, and mutations in lipopolysaccharide synthesis genes in two strains. Prophage deletion and induction were also noted in two strains, as well as mutations in several genes associated with virulence factors. Mutations in genes involved in susceptibility to conventional antibiotics were also identified in BIMs, with both decreased and increased antibiotic sensitivity to various antibiotics being observed. These findings may have implications for future applications of lytic phage therapy.

**IMPORTANCE:**

Lytic bacteriophages are viruses that infect and kill bacteria and can be used to treat difficult-to-treat bacterial infections, including biofilm-associated infections and multidrug-resistant bacteria. *Pseudomonas aeruginosa* is a bacterium that can cause life-threatening infections. Lytic bacteriophage therapy has been trialed in the treatment of *P. aeruginosa* infections; however, sometimes bacteria develop resistance to the bacteriophages. This study sheds light on the genetic mechanisms of such resistance, and how this might be harnessed to restore the sensitivity of multidrug-resistant *P. aeruginosa* to conventional antibiotics.

## INTRODUCTION

*Pseudomonas aeruginosa* is an aerobic gram-negative bacterium, considered to be an opportunistic pathogen. It is ubiquitous in the environment and capable of causing life-threatening sepsis, infective endocarditis, osteomyelitis, soft tissue infections, and respiratory infections (1). *P. aeruginosa* is of particular significance in cystic fibrosis-associated upper and lower respiratory tract infections. Chronic *P. aeruginosa* lung infection is associated with worsening lung function in cystic fibrosis (CF) patients. Phenotypic changes in the infecting strain over time can result in conversion to antibiotic-resistant mucoid phenotypes, which has been correlated with increased morbidity and mortality (2, 3). Patients with CF are also frequently affected by chronic rhinosinusitis (CRS), in which colonization of the paranasal sinuses with *P. aeruginosa* has been found to be a reservoir for recurrent lung infection (4–7).

Treatment of *P. aeruginosa* infections is complicated by high rates of single-drug and multi-drug resistance (8, 9). One potential treatment that is being investigated as an alternative to antibiotics is bacteriophage (phage) therapy. Virulent (lytic) bacteriophages are viruses that infect bacteria, replicate within the bacteria, and then cause bacterial lysis and death, releasing phage progeny in the process. Anti-*P*. *aeruginosa* bacteriophages have shown promise in several *in vitro* and *in vivo* pre-clinical studies as well as in controlled clinical studies (10–17). Some of the advantages of phage therapy over conventional antibiotics include activity against multi-drug resistant strains, the ability to target a specific bacterial species, and activity against biofilms (18–21).

However, several studies have described the emergence of phage-resistant bacterial isolates, also known as bacteriophage insensitive mutants (BIMs), following the treatment of *P. aeruginosa* biofilms with bacteriophages (22, 23). *De novo* resistance to bacteriophages may represent a potential barrier to the use of phage therapy in treating *P. aeruginosa* infections, but it may also be harnessed to decrease antibiotic resistance or as a target for the combination of phage with other anti-microbial therapies (24). Chan et al demonstrated that resistance to a phage that uses outer membrane porin M (OprM) of *P. aeruginosa* multi-drug efflux systems as a surface receptor produces an evolutionary trade-off in multi-drug resistant *P. aeruginosa*, resulting in increased sensitivity to conventional antibiotics (25).

In order to utilize anti-*P*. *aeruginosa* bacteriophage therapy in the most efficacious way, it is important that we understand the mechanisms underlying *de novo* phage resistance. The primary aim of this study was to examine genome variation in *P. aeruginosa* clinical isolates from the upper and lower respiratory tracts that have developed phage resistance following repeated exposure to a phage cocktail. An *in vitro* biofilm model was chosen as *P. aeruginosa* biofilms have been demonstrated in chronic respiratory infections and are associated with recurrent and treatment-recalcitrant disease (26–29).

## MATERIALS AND METHODS

### Bacterial strains

Two clinical *P. aeruginosa* strains (Ned 5 and USA 2) were isolated from the respiratory tract of CRS patients, as previously described (20). One clinical isolate (Ned 5) was from a CF patient. *P. aeruginosa* laboratory reference strain ATCC 15692 (PAO1) was obtained from the American Type Culture Collection (Manassas, VA, USA). All three strains exhibited a high degree of sensitivity to the bacteriophage cocktail (CT-PA) (20) when tested using the spot test assay described by Mazzocco et al. (20, 30). All strains were stored in 25% glycerol in nutrient broth at −80°C and grown on 1.5% nutrient agar or in nutrient broth (Oxoid, Hants, UK) at 37°C.

### Bacteriophage cocktail

Stocks of four anti-*P*. *aeruginosa* bacteriophages (Pa193, Pa204, Pa222, and Pa223) in phosphate-buffered saline with 0.01M magnesium sulfate (PBS + Mg) were supplied by AmpliPhi Biosciences (Brookvale, New South Wales, Australia). Pa193 and Pa204 are myoviruses, and Pa222 and Pa223 are podoviruses. All four phages have been characterized as strictly lytic by genome sequencing (unpublished data). Prior to each assay, the stock suspension of each bacteriophage was titrated against a selected *P. aeruginosa* bacterial strain using the soft agar overlay small drop assay, as described by Mazzocco et al. (30). Equal amounts of each bacteriophage were combined to form the bacteriophage cocktail (CT-PA) at a concentration of 1 × 10^8^ PFU/mL.

### Generation of bacteriophage-insensitive mutants

Biofilms of each *P. aeruginosa* strain were grown on separate clear polystyrene 96-well plates, as previously described (20). A total of 48 wells of 150 µL/well of 1.0 McFarland unit bacterial suspension diluted 1:10 into the nutrient broth, comprised of 2 blocks containing 24 wells each separated by PBS-filled wells, were inoculated in each plate. Plates were incubated on a gyratory mixer in a 37°C incubator for 7 days to allow biofilm formation, with 50 µL/well nutrient broth replenished at 24 h intervals. After the first 7 days, every 24 h, 50 µL of liquid media was aspirated from each well was immediately substituted with 50 µL of either 1 × 10^8^ PFU/mL CT-PA or PBS + Mg as a vehicle control. For each strain, one “block” of 24 wells was treated with CT-PA every 24 h, whereas the other “block” was treated with PBS + Mg every 24 h, for 7 consecutive days. Following 7 days of treatment, liquid contents of the wells were gently aspirated and discarded. Biofilms were gently washed with PBS twice to remove any remaining planktonic cells. The method for isolating bacteriophage-insensitive mutants (BIMs) was adapted from Buckling et al. (31). In total, 200 µL of 10^10^ PFU/mL CT-PA was spread evenly onto 1.5% nutrient agar plates, and excess liquid was allowed to evaporate. A sterile cotton swab was used to streak the bacteria from each row of wells in each CT-PA-treated “block” separately onto these agar plates. The same process was used to streak bacteria from the PBS + Mg-treated blocks onto 1.5% nutrient agar plates that had not been inoculated with phages. Agar plates were then incubated at 37°C overnight. Streaks that displayed bacterial growth without phage plaques were subcultured onto fresh 1.5% agar plates, and sensitivity or resistance to CT-PA was confirmed using the spot test assay.

### Bacterial DNA extraction and whole genome sequencing

Bacterial genomic DNA extraction and purification were performed as previously described (20). The quality and concentration of the purified DNA were assessed using the NanoDrop 2000 spectrophotometer (Thermo Fisher Scientific, Waltham MA, USA), gel electrophoresis, and the Invitrogen Qubit 3 fluorometer (Thermo Fisher Scientific). For short-read sequencing, DNA samples were sent to two external laboratories (Australian Genome Research Facility, Melbourne, Australia and SA Pathology, Adelaide, Australia) for whole genome sequencing on the MiSeq and NextSeq platforms (Illumina) with 250 bp and 150 bp paired-end reads, respectively, to a depth of 100 × *P*. *aeruginosa* PAO1 reference genome length. Long-read sequencing was performed using an Oxford Nanopore MinION mk1c (Oxford Nanopore, Oxford, UK) using R9.4.1 MinION flowcells (Oxford Nanopore Technology) with the SQK-RBK 110.96 Rapid Barcoding Kit. For one isolate (vehicle-treated USA 2), purification and size selection using AMPure XP magnetic beads (Beckman Coulter, Brea CA, USA) was required to obtain satisfactory long-read sequencing. The manufacturer’s protocol was followed, with the exception of an AMPure XP to sample volume ratio of 0.65 to select for long DNA fragments, and the final elution was performed into nuclease-free water (32). Base-calling was conducted with Guppy v6.2.11 super accuracy mode using the “dna_r9.4.1_450bps_sup.cfg” configuration (Oxford Nanopore Technology).

### Genome assembly

*P. aeruginosa* assemblies were created with a long-read first approach using Trycycler (33). Read sets were first subsampled into 12 batches. Each subset was assembled with Flye v2.9.1 with “—nano-hq” specified (34). All assemblies were then clustered using “trycycler cluster.” For each isolate, the cluster corresponding to the chromosome (ie, with a length of approximately 6.2 MB) was identified, with a consensus sequence derived from this cluster using the “trycycler msa,” “trycycler partition,” and “trycycler consensus” commands. The resulting chromosomes were polished with long reads first using Medaka v1.7.0 (Oxford Nanopore Technologies, 2022), then with short reads using Polypolish v0.5.0 (35). After the first round of polishing, the chromosomes were reoriented to begin at the putative dnaA gene using the customized python program dnaapler (36). Chromosomes were then polished a second time with Polypolish, followed by POLCA (37).

### Variant calling and annotation

A customized pipeline was created to conduct variant calling and annotation using Snakemake (38). All assemblies were annotated with Bakta v1.6.1 (39). All chromosomes were MLST-typed using mlst (available at https://github.com/tseemann/mlst) and assigned to clonal complexes using PubMLST (40). Antimicrobial resistance and virulence genes were identified by screening all isolate contigs through the Comprehensive Antibiotic Resistance Database (41) and Virulence Factor Database (42) using ABRicate, v1.0.1 (available at https://github.com/tseemann/abricate). The putative prophage deletion in PAO1, and the induced prophage in Ned 5 were annotated with Pharokka v1.2.0 (43). Specifically, coding sequences (CDS) were predicted with PHANOTATE (44), with functional annotation generated by matching each CDS to the PHROGs database (45) using MMseqs2 (46). The contig was also matched to the closest hit in the INPHARED database (47) using mash (48). All small variants (single nucleotide polymorphisms [SNPs] and small insertions and deletions) between control and BIM isolates were called using Snippy v4.6.0 (available at https://github.com/tseemann/snippy). The raw FASTQ short reads from the Timepoint BIM isolate were compared against the genbank file of the assembled Timepoint Control isolate for each bacterial strain as a reference. All larger structural differences were called using Nucdiff v2.0.3 (49) by comparing the chromosome assembly of the Control isolate against the BIM isolate and also using Sniffles v2.0.7 (50) to compare the long reads from the BIM isolate against the assembly of the control. All putative structural deletions and small nucleotide variants were manually screened by mapping all BIM long reads to the Control assembly using minimap2 v2.24 (51) specifying “-ax map-ont” and then sorting the resulting BAM file using samtools (52). All putative insertions and duplications were manually screened by mapping the Control long reads to the BIM assembly in the same way. All pileups were visualized using IGV genome browser v2.10.2 (53). All genomic maps were made with Clinker v0.0.27 (54).

### Prophage induction

The prophage induction in Ned 5 was identified using a customized version of hafeZ (55) applied on Nanopore long reads, which can be found at https://github.com/gbouras13/hafeZ/tree/ont. This modification works by identifying all areas of increased coverage compared with the rest of the chromosome and finding all reads with both primary and supplementary mappings in the same area (corresponding to the start and the end of the induced prophage). If there are at least five reads spanning the start and the end of the region, it is denoted as an induced prophage. Web BLAST was used to compare the Ned 5 prophage against Phage PBD44 using default parameters (56).

### N-acetylmuramoyl-L-alanine amidase *amiB* protein structure visualization

The predicted protein structure for the *amiB* protein with accession AF-A0A0C7AR16-F1-v4 was downloaded from the Alphafold database (https://alphafold.ebi.ac.uk/entry/A0A0C7AR16) (57). The protein structure was visualized with ChimeraX v1.7.1 (58).

### Minimum inhibitory concentration of antibiotics

The minimum inhibitory concentration (MIC) of different antibiotics including tetracycline, chloramphenicol, ciprofloxacin, meropenem, and imipenem against planktonic forms of the bacterial isolates was assessed using the microdilution method as described by the Clinical and Laboratory Standard Institute (CLSI) (59). The bacterial isolates were streaked onto nutrient agar. Single colonies of bacteria were resuspended in nutrient broth to adjust to 0.5 ± 0.1 McFarland units (approximately 1.5 × 10^8^ Colony-Forming Units [CFU]/mL). Then, a dilution of bacteria 1:100 in nutrient broth was prepared, and 96-well microtiter plates were inoculated with 50 µL of the diluted bacterial suspensions, followed by 50 µL of different concentrations of antibiotics (antibiotic solvents were water, 95% ethanol, 0.1N hydrochloric acid, water and water for tetracycline, chloramphenicol, ciprofloxacin, meropenem, and imipenem, respectively) and incubated at 37°C for 18–24 h. The MIC was reported as the lowest concentration at which no visible turbidity was shown.

### Minimum biofilm eradication concentration of antibiotics

The minimum biofilm eradication concentration (MBEC) of antibiotics was determined as previously described (60). Briefly, one colony of bacteria was resuspended in 0.9% saline to adjust to 1.0 ± 0.1 McFarland units (approximately 3 × 10^8^ CFU/mL). The suspension was diluted 1:15 in tryptone soy broth (TSB), and black 96-well plates (Costar, Corning Incorporated, Corning, U.S.) were inoculated with 150 µL of diluted bacterial suspensions. Suspensions were incubated at 37°C for 48 h on a rotating platform at 70 rpm in a 5% CO_2_ incubator. Established biofilms were washed twice with phosphate-buffered saline (PBS) and exposed to different concentrations of antibiotics in TSB for 24 h at 37°C on a rotating platform at 70 rpm, in a 5% CO_2_ incubator. Biofilms were washed twice with PBS followed by measurement of bacterial viability via the resazurin assay (Life Technologies, Scoresby, Australia).

### Resazurin assay

Briefly, 200 µL of a freshly prepared 10% resazurin dilution in TSB was added and incubated at 37°C on a rotating platform at 70 rpm in a 5% CO_2_ incubator, protected from light. The fluorescent intensity was measured after 9 h on a FLUOstar OPTIMA plate reader (BGM Labtech Gretenberg, Germany) at λ excitation = 530 nm/λ emission = 590 nm. Biofilm eradication percentage (BE%) was quantified according to below Eq.


$$BE\%=100-\left(\frac{F_{T}}{F_{C}}\times 100\%\right)$$

Antimicrobial activity of antibiotics is expressed as BE%, where *F*_*C*_ is the fluorescence of the untreated control biofilm (100% biofilm viability), and *F*_*T*_ is the fluorescence observed in the treated biofilm. Both *F*_*C*_ and *F*_*T*_ were subtracted from background fluorescence (broth). Biofilm eradication studies were performed as three independent experiments with two wells per treatment. Minimum biofilm eradication concentration (MBEC) was defined as the lowest antibiotic concentration where a mean BE% of at least 50% was measured.

## RESULTS

### Generation of BIMs and phage sensitivity testing

Multiple BIMs were isolated from all CT-PA-treated biofilm plates. One BIM was also isolated from vehicle-treated PAO1 biofilms. No BIMs were detected in any of the other vehicle-treated biofilms. A single CT-PA-treated BIM from each bacterial strain was selected for whole genome sequencing, in order to optimize sequencing read depth. All the sequenced BIMs were confirmed as being resistant to CT-PA by the complete absence of spots and plaques on the spot test assay, tested up to a maximum concentration of 1 × 10^8^ PFU/mL CT-PA. All sequenced vehicle-treated isolates were confirmed as being sensitive to CT-PA on the spot test assay, with measured phage titers of 3 × 10^7^, 4 × 10^8^, and 6 × 10^7^ PFU/mL from a stock solution of 1 × 10^8^ PFU/mL of CT-PA, for PAO1, Ned 5 and USA 2 vehicle-treated isolates, respectively.

### Whole genome sequencing and genome assembly

A complete circular chromosome assembly was achieved for all isolates, with mean sequencing long read depth ranging from 13× to 27× coverage. Lengths varied from 6,216,926 bp to 6,301,445 bp (Table 1).

**TABLE 1:** Length of assembled genomes and mean long read depth for vehicle control-treated isolate and BIM for all three strains tested

Strain	Vehicle control-treated isolate		BIM isolate	
	Length (bp)	Mean long read depth	Length (bp)	Mean long read depth
PAO1	6,259,812	27	6,216,926	24
Ned 5	6,279,641	14	6,291,135	13
USA 2	6,301,476	20	6,301,489	27

### Variant calling and annotation

A summary of findings from the comparative genomic analysis between BIMs and vehicle-treated control isolates in each strain is shown in Table 2.

**TABLE 2:** Summary of the findings from the comparative genomic analysis between BIMs and vehicle-treated control isolates in each of the three *P*. *aeruginosa* strains studied

*P. aeruginosa* strain	Genomic variants identified in BIM compared with vehicle-treated control	
	Structural variants	Single nucleotide variants (SNVs)
PAO1	42,846 bp deletion, corresponding to a likely prophage region	7 SNVs (three in coding sequences), including one deletion in *rfaB*, a lipopolysaccharide synthesis gene
Ned 5	Prophage induction, containing *bstA* gene encoding for abortive infection system protein 11,426 bp tandem duplication containing *fepA* gene encoding for TonB-dependent receptor	82 SNVs (19 in coding sequences), including one deletion in *rfaB* lipopolysaccharide synthesis gene
USA 2	No structural variants identified	Four SNVs (one in a coding sequence), including a single nucleotide variant in the N-acetylmuramoyl-L-alanine amidase *amiB*

#### PAO1

**Fig 1:**
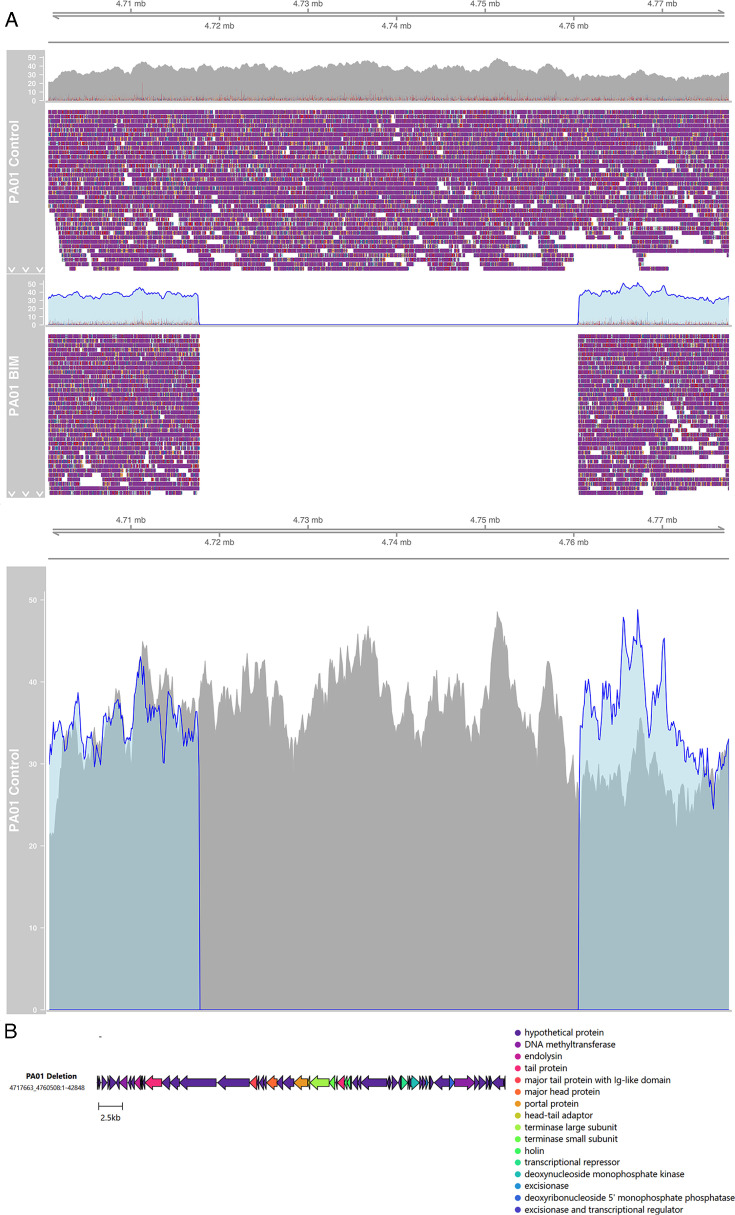
(**A**) All long reads of PAO1 BIM mapped onto the PAO1 Control genome, visualized using IGV-style pileups. The upper track represents the long read coverage and the lower (colored) part visualizes the read alignments. The gap in the alignment, indicating the deletion, can be clearly seen in the empty middle of the plot, where there are no reads. (**B**) Gene map of the deleted region, colored by predicted Pharokka PHROG annotation.

A large deletion of 42,846 bp from nucleotide 4717663 to 4760508 was identified in the PAO1 phage-treated BIM compared with the PAO1 vehicle control-treated isolate (Fig. 1A). This region corresponds to a likely prophage region, identified according to PhiSpy. On the basis of this purported function, the region was extracted and reannotated with Pharokka v1.2.0. According to the Pharokka annotations, there are a large number of phage genes in this deletion (Fig. 1B; Table S1). Of the 60 CDS identified by Pharokka, 18 have known functions, including large and small terminase subunits, an endolysin, holin, and head packaging proteins, whereas 43 of 60 could be mapped to a PHROG group, indicating that they are likely phage-encoded genes. However, there was no close hit to the INPHARED database, suggesting that this region of the genome may perhaps be a cryptic prophage (61).

In addition to this structural variant, seven small nucleotide variants (SNVs) were identified by Snippy in the PAO1 phage-treated BIM compared with the PAO1 vehicle control-treated isolate (Table 3). Of these variants, three were contained within coding sequences, all frameshift effects. In particular, there was a small deletion in the *rfaB* glycosyltransferase gene, which is involved in lipopolysaccharide synthesis (62).

**TABLE 3:** Each small variant found in the PAO1 BIM compared with the vehicle-treated control according to Snippy^*a*^

Position	Variant type	REF	ALT	Evidence	Coding sequence	Effect	Gene	Product	Uniref90 accession (Bakta)
1641001	INS	C	CT	CT:13 C:0	No				
1641345	INS	C	CT	CT:20 C:0	No				
2739010	INS	A	AG	AG:56 A:1	Yes	Frameshift c.2446dupG p.Asp816fs		Filamentous hemagglutinin N-terminal domain-containing protein	UPI00066C6EF2
2830423	INS	A	AG	AG: 72 A:0	Yes	Frameshift c.2232dupG p.Ser745fs		Condensation domain-containing protein	UPI000A704DFD
4468696	INS	C	CG	CG:5 C:0	No				
5570732	DEL	TCG	T	T:91 TCG:7	Yes	Frameshift c.841delCG p.Glu281fs	*rfaB*	RfaB glycosyltransferase	A0A2R3IUW8
5750712	INS	C	CT	CT:54 C:1	No				

^*a*^ For variant type, “INS” refers to insertion while “DEL” refers to deletion. “REF” refers to the sequence in the untreated control. “ALT” refers to the sequence in the BIM. Evidence refers to the depth of coverage of both the REF and ALT alleles.

#### Ned 5

**Fig 2:**
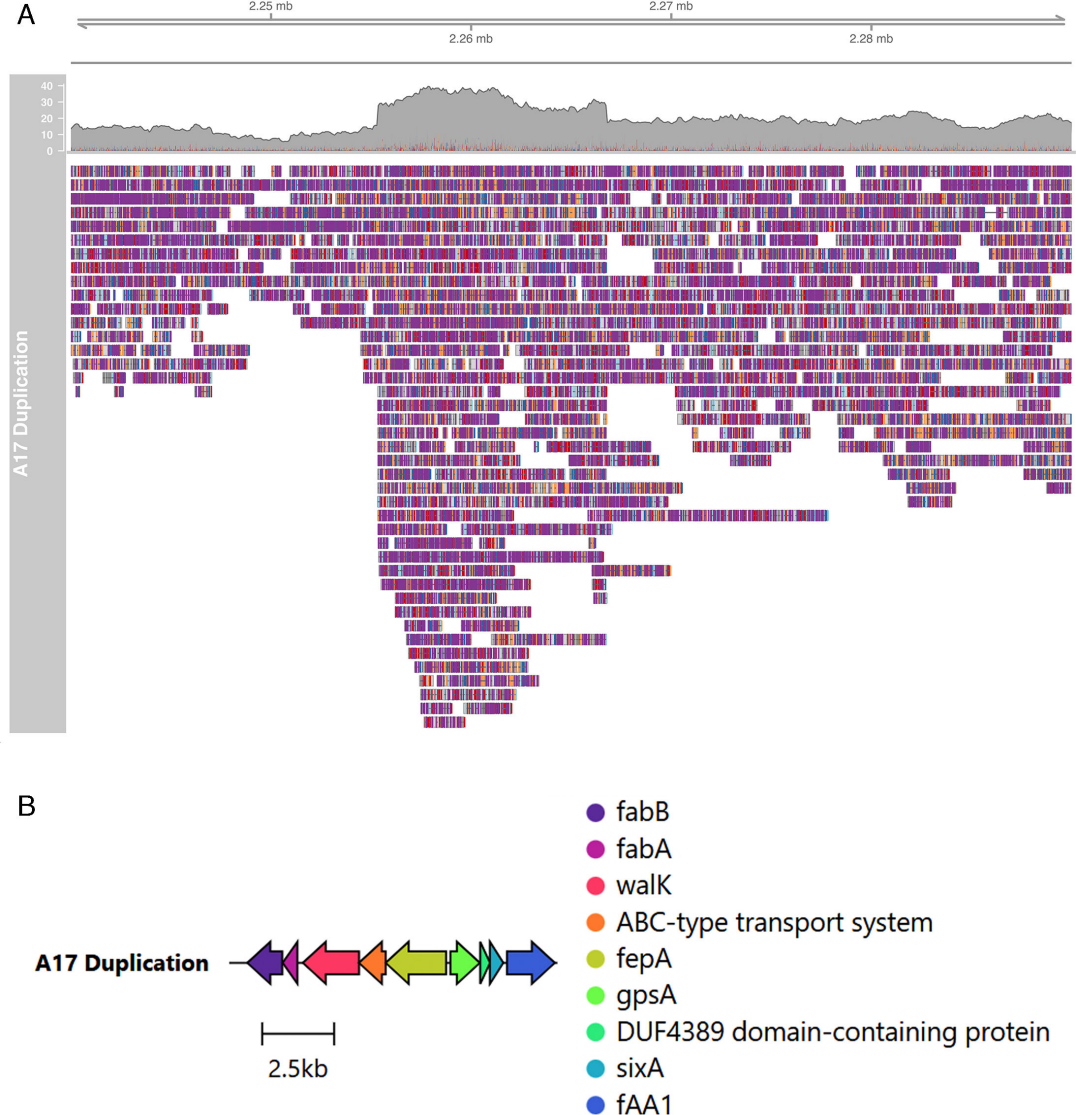
(**A**) All long reads of the Ned 5 BIM mapped onto the Ned 5 Control genome, visualized in IGV-style pileups between bases 2,240,000 and 2,290,000 bp. The upper track represents the coverage plot, and the lower part visualizes the read alignments. The tandem duplication can be seen in the middle, where the area of increased coverage with clipped reads on both sides indicates a repeated region. (**B**) Gene map of the duplicated region.

A large tandem duplication of 11,426 bp was identified by both Sniffles and Nucdiff in the Ned 5 phage-treated BIM compared with the Ned 5 vehicle control-treated isolate (Fig. 2A). The repeated region was comprised of two identical repeated sequences, each containing nine CDS, of which eight are annotated with a known function (Fig. 2B; Table 4). Notably, this region contains a TonB-dependent outer membrane receptor, FepA, of length 702 amino acids (AA). FepA has been shown to be involved in iron transport from the extracellular space into the periplasm in *Escherichia coli* (63).

**TABLE 4:** Each CDS in the duplicated region in the Ned 5 BIM compared with the untreated control, identified by Nucdiff and Sniffles

Start (BIM)	End (BIM)	Strand^*a*^	Gene	Uniref100 accession (Bakta)	Description
2267424	2268641	-	*fabB*	UPI0015D575E4	Beta-ketoacyl-ACP synthase I
2268653	2269168	-	*fabA*	A6V7I3	3-Hydroxyacyl-[acyl-carrier-protein] dehydratase FabA
2269357	2271312	-	*walK*	A0A8G4Y5L2	Hybrid sensor histidine kinase/response regulator
2271309	2272238	-		A0A1C7BUR4 (Uniref90)	ABC-type uncharacterized transport system 2C periplasmic component
2272239	2274347	-	*fepA*	A0A367MDX1	TonB-dependent receptor
2274498	2275520	+	*gpsA*	A0A6B1YDK9	NAD(P)H-dependent glycerol-3-phosphate dehydrogenase
2275534	2275875	+		A0A072ZI60	DUF4389 domain-containing protein
2275872	2276336	+	*sixA*	A0A069PZD3	Phosphohistidine phosphatase SixA
2276463	2278130	+	*fAA1*	A0A8G2QJS1	AMP-binding protein

^*a*^ For strand, “+” refers to the positive DNA strand, and “-” refers to the negative DNA strand.

**Fig 3:**
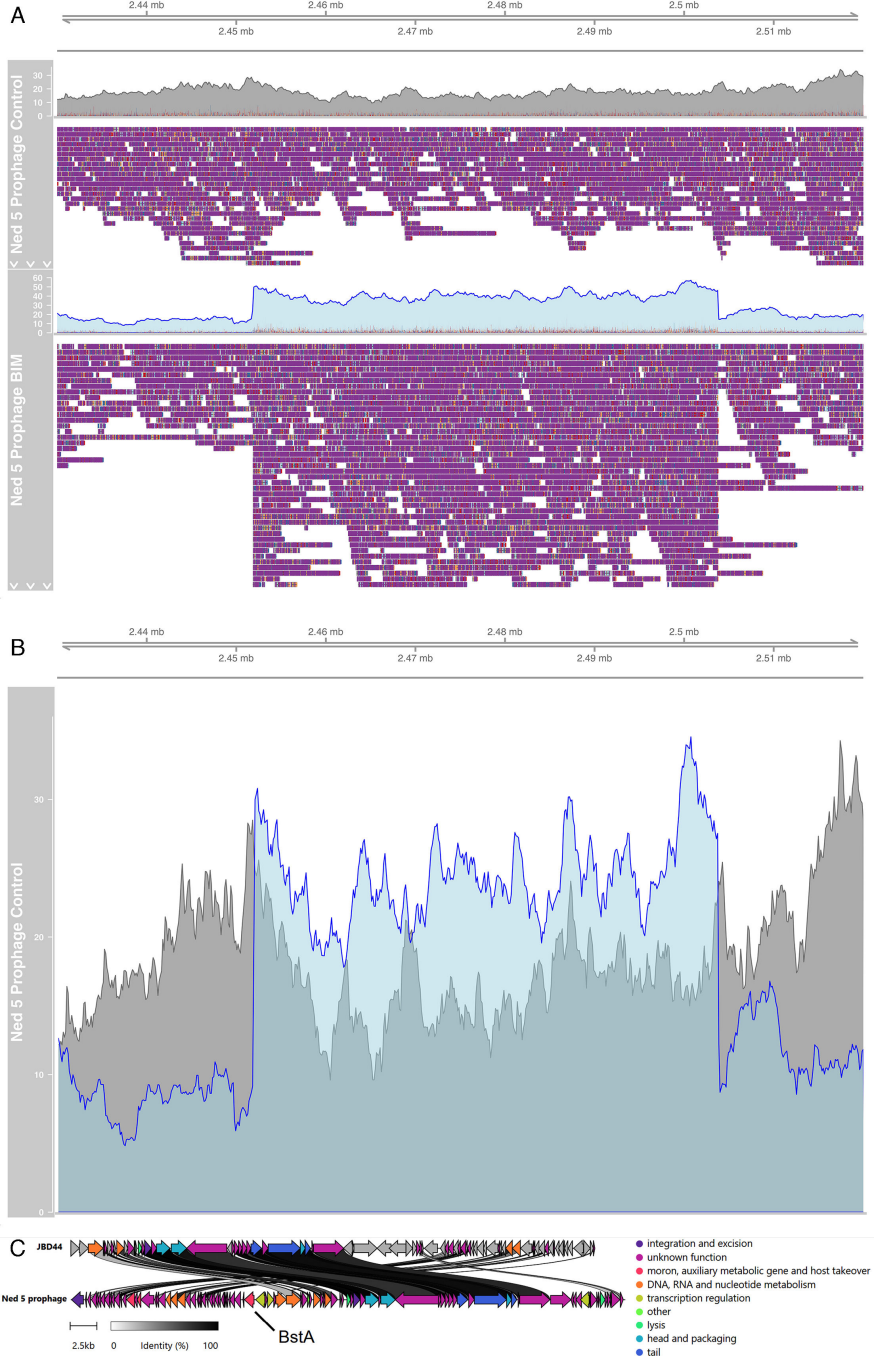
(**A**) All long reads of the Ned 5 Control and BIM mapped onto the control genome, visualized in IGV-style pileups between bases 2430000 bp and 2520000 bp. The upper track represents the coverage plot, and the lower part visualizes the read alignments. The even coverage of the Control reads (shaded in gray) indicates the prophage in this region was not induced in the Control isolate. The induced prophage can be seen in the middle of the BIM pileup, where the area of increased coverage of the BIM reads (in light blue) with clipped reads on both sides indicates an induced prophage, verified by hafeZ. (**B**) Coverages of the Ned 5 Control and BIM reads mapped onto the Control genome. The Control reads are in gray; the BIM reads are in blue. (**C**) Gene synteny plot of induced prophage (bottom) against its closest relative *Pseudomonas* phage JBD44 (top). The BstA gene is highlighted in the Ned 5 gene map.

There was also evidence of prophage induction in the Ned 5 BIM, which can be seen in the increased coverage of the prophage region from 2451890 to 2503760 bp (Fig. 3A). This induction was not observed in the Ned 5 vehicle control-treated isolate (Fig. 3B). Annotation of this prophage with Pharokka revealed that it has 95 CDS with 24 of known function. Its closest relative in the INPHARED database is Pseudomonas Phage JBD44 (Refseq NC_030929.1). However, according to a follow-up BLAST search, the Ned 5-induced prophage only shared 45% coverage homology (96.7% identity over this region) (Fig. 3C). Of note, this induced prophage carries the BstA abortive infection system protein, which is known to confer a defensive mechanism against exogenous phages, by mediating abortive infection (64).

In addition, 82 SNVs were detected by Snippy in the Ned 5 phage-treated BIM compared with the Ned 5 vehicle control-treated isolate (Table S2), 19 of which were in coding sequences (Table 5). No CDS containing multiple SNVs was identified. A small deletion in the *rfaB* glycosyltransferase gene was also identified in the Ned 5 BIM (also found to be mutated in the PAO1 BIM).

**TABLE 5:** CDS small nucleotide variants (SNVs) found in the Ned 5 BIM compared with the vehicle-treated control according to Snippy^*a*^

Position	Variant type	REF	ALT	Evidence	Effect	Gene	Product	Uniref90 accession (Bakta)
96662	INS	C	CG	CG:34 C:0	frameshift_variant c.608dupC p.Arg204fs		Type VI secretion system-associated FHA domain protein TagH	UPI00053D4030
336483	INS	C	CG	CG:92 C:1	frameshift_variant c.173dupC p.Leu59fs		Hypothetical protein	None
658908	INS	A	AGGG	AGGG:52 A:0	conservative_inframe_insertion c.89_91dupCCC p.Pro30dup	*pdxA*	4-Hydroxythreonine-4-phosphate dehydrogenase PdxA	Q9I5U4
1815592	SNP	A	G	G:64 A:0	missense_variant c.1463A > G p.Gln488Arg	*czcO*	FAD-containing monooxygenase EthA	A0A080VSN6
1830880	INS	C	CT	CT:57 C:1	frameshift_variant c.1132dupA p.Arg378fs	*mltA*	Transglycosylase	A0A2R3IXK2
2035180	INS	A	AC	AC:87 A:0	frameshift_variant c.705dupC p.Pro237fs		UvrD-helicase domain-containing protein	UPI00066CA611
2096480	INS	A	AG	AG:74 A:0	frameshift_variant c.1806dupG p.Pro603fs		Phosphorelay protein LuxU	Q5E3S0 (Uniref50)
3115706	INS	G	GT	GT:45 G:0	frameshift_variant c.4775dupA p.Asn1592fs	*entF*	EntF, seryl-AMP synthase component of non-ribosomal peptide synthetase	A0A643EKV7
3133701	INS	A	AC	AC:77 A:0	frameshift_variant c.2232dupG p.Ser745fs		Amino acid adenylation domain-containing protein	UPI000F51FCBD
3242350	SNP	C	T	T:62 C:0	missense_variant c.1226C > T p.Thr409Ile	*baeS*	Two-component sensor histidine kinase	A0A069PZI5
3477161	SNP	G	A	A:73 G:0	missense_variant c.851G > A p.Arg284His		RNA-splicing ligase RtcB	A0A0P0AID0
3689752	INS	A	ACC	ACC:59 A:0	intragenic_variant n.3689752_3689753insCC	*aspB*	Aminotransferase	A0A073A5B3 (Uniref100)
3690082	INS	T	TC	TC:69T:0	frameshift_variant c.285dupC p.Gly96fs		Hypothetical protein	None
4255141	INS	C	CG	CG:48 C:1	frameshift_variant c.1204dupC p.Arg402fs		Tfp pilus assembly protein FimV	A0A485FKG2
4417443	INS	G	GC	GC:51 G:0	frameshift_variant c.1383dupG p.Leu462fs	*opmE*	Outer membrane efflux protein	A0A509JP07
4777540	INS	T	TA	TA:62T:0	frameshift_variant c.967_968insA p.Val323fs		ABC transporter substrate-binding protein	A0A080VS54
4823740	SNP	A	G	G:54 A:0	synonymous_variant c.426T > C p.Asn142Asn	*narK*	MFS transporter	A0A069Q5D4
4823749	SNP	A	G	G:59 A:0	synonymous_variant c.417T > C p.Gly139Gly	*narK*	MFS transporter	A0A069Q5D4
5038318	INS	C	CG	CG:38 C:0	frameshift_variant c.709dupC p.Arg237fs		Hypothetical protein	A0A3M5ER77
5632533	DEL	ACTTGGT	A	A:69 ACTTGGT:0	conservative_inframe_deletion c.556_561delACCAAG p.Thr186_Lys187del	*rfaB*	Glycosyltransferase	A0A2R3IUW8
6112384	SNP	C	G	G:73 C:0	missense_variant c.533C > G p.Ala178Gly	*fadH2*	Sarcosine oxidase subunit alpha	UPI0003B9BA99 (Uniref100)

^*a*^ For variant type, “INS” refers to insertion, “DEL” refers to deletion, and “SNP” refers to single nucleotide polymorphism. “REF” refers to the sequence in the untreated control, “ALT” refers to the sequence in the BIM. Evidence refers to the depth of coverage of both the REF and ALT alleles.

#### USA 2

**Fig 4:**
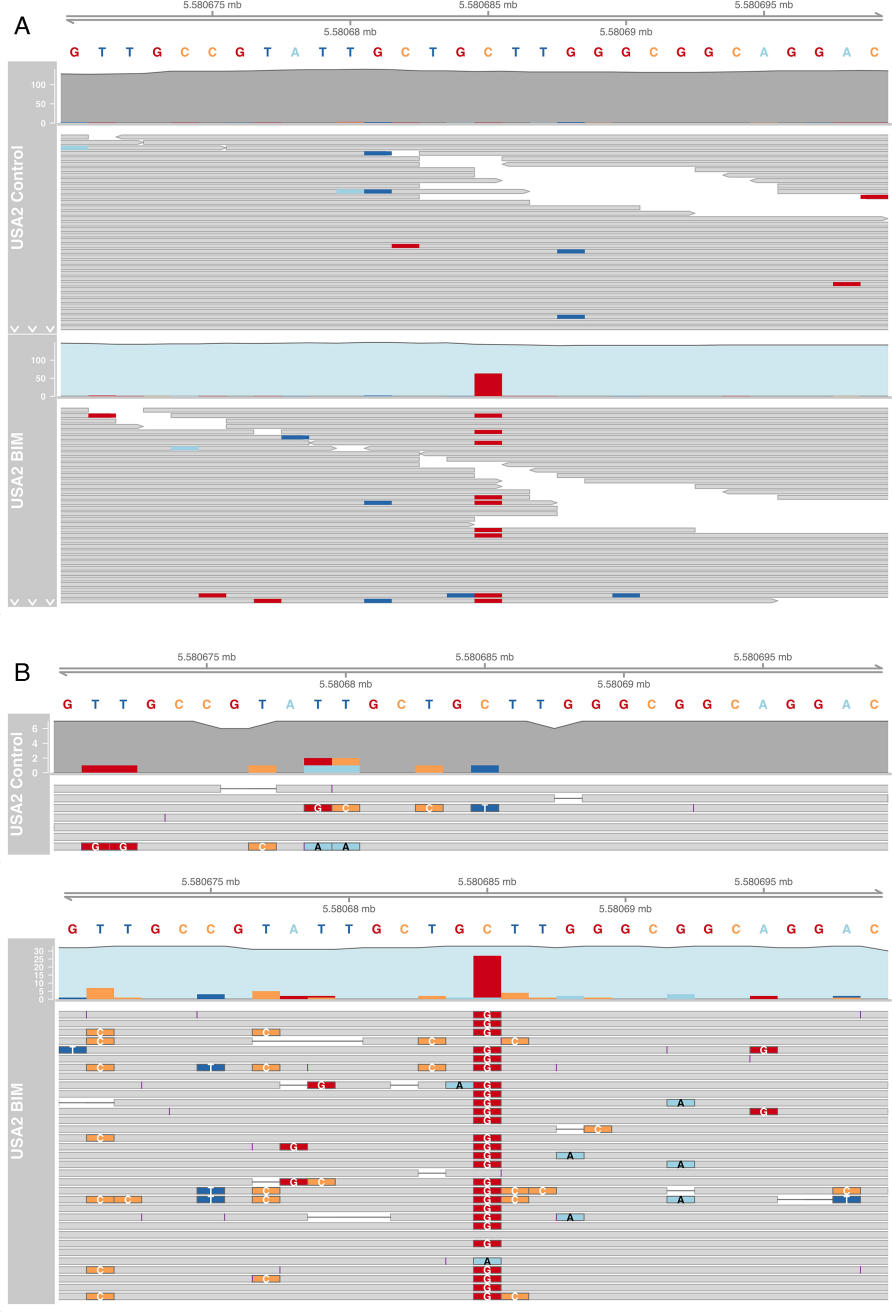
(**A**) All short reads and (**B**) long reads of the USA 2 Control and BIM mapped onto the USA 2 Control genome, visualized in IGV-style pileups between bases 5580670 bp and 5580700 bp. The top track shows the reference sequence. The upper track for each read set represents the coverage plot, and the lower pileup visualizes the read alignments. The Control read coverage is indicated in gray, and the BIM read coverage in light blue. At position 5580685 bp, reads mapping to the reference allele (“C”) are indicated in gray, whereas reads mapping to the alternative allele (‘G’) are indicated in red. For the short reads, the alternative allele frequency is approximately 45%, whereas for the long reads, it is approximately 80%.

There were no structural variants detected upon comparison of the USA 2 phage-treated BIM with its vehicle control-treated counterpart. However, USA 2 harbored a number of SNVs in the BIM compared with the vehicle control-treated isolate. Four likely SNVs were identified in the USA 2 phage-treated BIM compared with the USA 2 vehicle control-treated isolate (Table 6), one of which was in a coding sequence. This SNV was located in the *amiB* N-acetylmuramoyl-L-alanine amidase gene, with length 475 amino acids. This SNV showed an allele frequency of approximately 45% in the short read data and 80% in the long-read data (Fig. 4A and B). Specifically, the SNV was annotated as a missense variant at nucleotide 366, leading to a change from Lysine to Asparagine in amino acid 122.

**TABLE 6:** CDS small nucleotide variants (SNVs) found in the USA 2 BIM compared with the vehicle-treated control according to Snippy^*a*^

Position	Variant type	REF	ALT	Evidence	Coding sequence	Effect	Gene	Product	Uniref100 accession (Bakta)
1084370	INS	A	AG	AG:10 A:0	No				
1525492	INS	T	TTG	TTG:37T:0	No				
5154832	INS	C	CT	CT:9 C:0	No				
5580685	SNP	C	G	C:80 G:65 (short), C:6 G:26 (long)	Yes	missense_variant c.366C > G p.Lys122Asn	*amiB*	N-acetylmuramoyl-L-alanine amidase AmiB	A0A0C7AR16

^*a*^ For variant type, “INS” refers to insertion, “DEL” refers to deletion, and “SNP” refers to single nucleotide polymorphism. “REF” refers to the sequence in the vehicle-treated control, “ALT” refers to the sequence in the BIM. Evidence refers to the depth of coverage of both the REF and ALT alleles.

**Fig 5:**
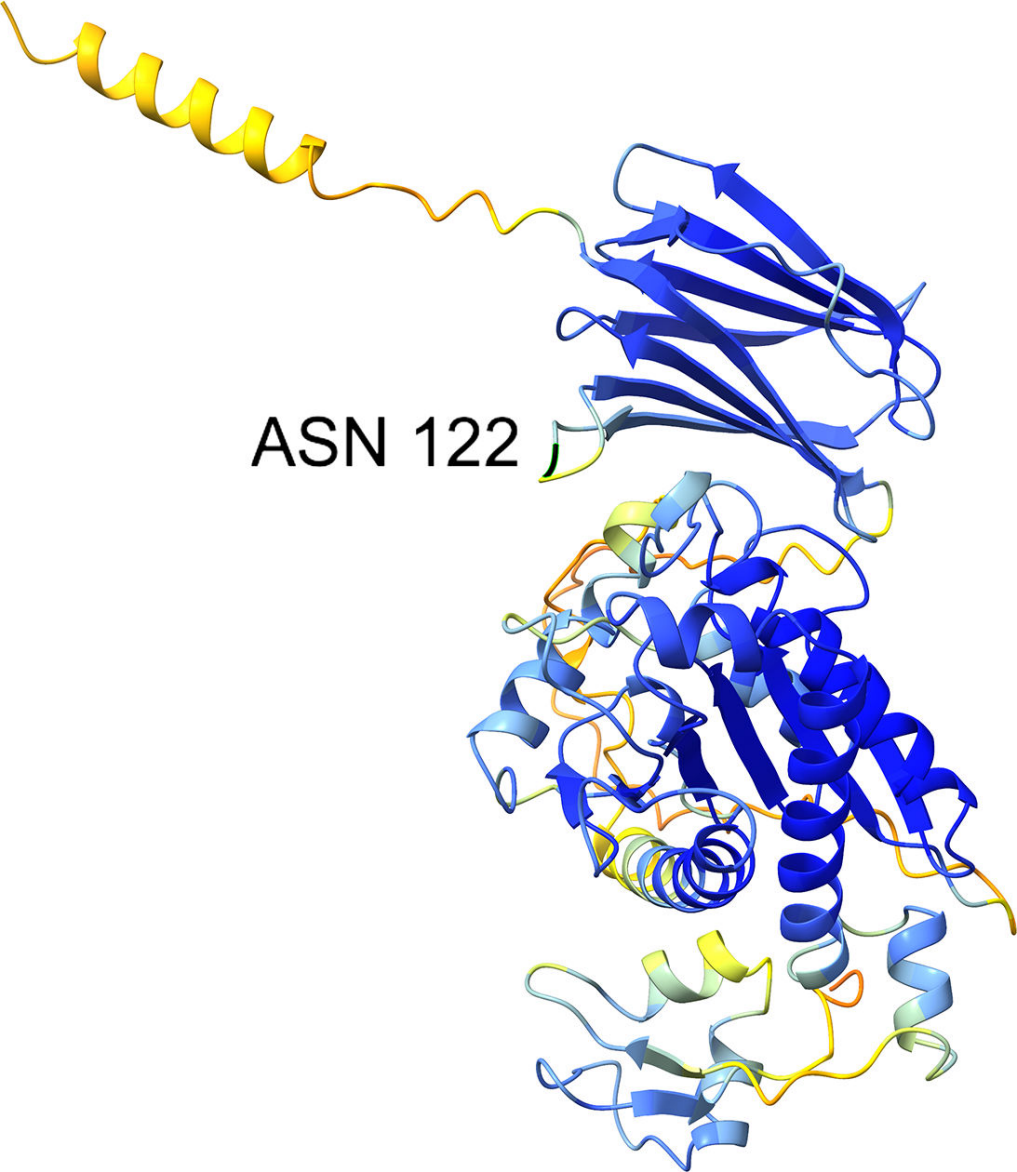
Alphafold database predicted structure of AmiB N-acetylmuramoyl-L-alanine amidase (accession AF-A0A0C7AR16-F1-v4). Each residue is colored using the ChimeraX “alphafold” color palette, other than the variant Asparagine residue (residue 122) colored black and bordered light green for contrast. Blue resides are very confidently predicted with a predicted local distance difference test (pLDDT) value of over 90, light blue is confidently predicted with a pLDDT value between 70 and 90, yellow indicates 50–70 with medium confidence, and orange indicates low confidence (<50).

Structural analysis of the predicted AmiB protein structure from Alphafold database (57) (accession: AF-A0A0C7AR16-F1-v4) revealed that overall the structure was confidently predicted (average predicted local distance difference test [pLDDT] value of 83.9) and that the Lysine to Asparagine change occurs within a beta-hairpin motif (Fig. 5).

### Minimum inhibitory concentration (MIC) of antibiotics

The results of the MIC testing for all isolates are shown in Table 7. Several differences in susceptibility to conventional antibiotics between phage-treated and vehicle control-treated isolates were detected in all three strains. The phage-treated PAO1 isolate displayed increased susceptibility to tetracycline (2-fold increase), chloramphenicol (4-fold increase), and meropenem (2-fold increase). Imipenem susceptibility was increased compared with the parent strain, but less than that of the vehicle-treated control isolate. The phage-treated Ned 5 isolate displayed increased susceptibility to chloramphenicol (8-fold increase) and ciprofloxacin (2-fold increase), compared with both the parent and vehicle control-treated Ned 5 isolates. When comparing the phage-treated and vehicle control-treated USA 2 isolates, there was a similar pattern of carbapenem susceptibility as in PAO1, with the phage-treated isolate being four times more susceptible to meropenem than the control-treated isolate (but similar meropenem susceptibility to the parent strain), and 32 times less susceptible to imipenem compared with the control-treated isolate. There were also some differences in carbapenem and chloramphenicol susceptibility between the parent strains and their respective vehicle control-treated isolates.

**TABLE 7:** MIC of various conventional antibiotics against parent, vehicle control-treated, and phage cocktail (CT-PA)-treated isolates of each of the three *P*. *aeruginosa* strains^*a*^

Bacterial isolate	MIC (µg/mL)				
	Tetracycline	Chloramphenicol	Ciprofloxacin	Meropenem	Imipenem
PAO1 parent	8	32	0.25	0.25	16
PAO1 vehicle control-treated	**8**	**32**	0.25	**1**	**2**
PAO1 CT-PA-treated	**4**	**8**	0.25	**0.5**	**4**
Ned 5 parent	8	4	32	32	32
Ned 5 vehicle control-treated	8	**16**	**32**	4	1
Ned 5 CT-PA-treated	8	**2**	**16**	4	1
USA 2 parent	8	16	128	0.125	8
USA 2 vehicle control-treated	8	**8**	128	**0.5**	**1**
USA 2 CT-PA-treated	8	**16**	128	**0.125**	**32**

^*a*^ Differences in antibiotic sensitivity between the vehicle control-treated isolate and phage-treated BIM for each strain are shown in bold typeface.

### Minimum biofilm eradication concentration (MBEC) of antibiotics

The minimum biofilm eradication concentrations of the conventional antibiotics tested against the parent, phage-treated, and vehicle control-treated isolates of each of the three *P*. *aeruginosa* strains are displayed in Table 8. For PAO1, both the vehicle-treated control and phage-treated isolate displayed increased MBEC for all antibiotics, except ciprofloxacin, compared with the parent isolate. There were no differences in MBEC of the antibiotics tested between the vehicle-treated control and the phage-treated PAO1 isolates. For Ned 5, the MBEC of chloramphenicol was decreased 2-fold in the phage-treated isolate compared with the parent and vehicle-treated control isolates. For USA 2, the vehicle-treated control isolate displayed a decreased MBEC of tetracycline and chloramphenicol (2-fold decrease) compared with both the parent and phage-treated isolates.

**TABLE 8:** MBEC of various conventional antibiotics against parent, vehicle control-treated, and phage cocktail (CT-PA)-treated isolates of each of the three *P. aeruginosa* strains^*a*^

Bacterial isolate	MBEC (µg/mL)				
	Tetracycline	Chloramphenicol	Ciprofloxacin	Meropenem	Imipenem
PAO1 parent	256	128	512	32	32
PAO1 vehicle control-treated	>512	256	512	>512	>512
PAO1 CT-PA-treated	>512	256	512	>512	>512
Ned 5 parent	>512	512	>512	>512	>512
Ned 5 vehicle control-treated	>512	**512**	512	>512	>512
Ned 5 CT-PA-treated	>512	**256**	512	>512	>512
USA 2 parent	>512	256	512	>512	>512
USA 2 vehicle control-treated	**512**	**128**	512	>512	>512
USA 2 CT-PA-treated	**>512**	**256**	512	>512	>512

^*a*^ Differences in antibiotic sensitivity between the vehicle control-treated isolate and phage-treated BIM for each strain are shown in bold typeface.

## DISCUSSION

In this study, BIMs were generated through repeated exposure of *P. aeruginosa* biofilms to a phage cocktail, over a treatment period of 7 days. We identified a variety of genomic variants in BIMs compared with the corresponding vehicle-treated control isolate for the three strains tested. Large structural variants along with additional small variants were found in two strains (PAO1 and Ned 5), whereas the third strain (USA 2) harbored multiple small variants. We postulate that the variation in genomic mutations between strains is the result of the complex interaction between a cocktail of four distinct bacteriophages and the host strains, which have varying susceptibility to each individual phage in the cocktail (20).

Mutations involving TonB-dependent receptors were found in the Ned 5 BIM. TonB-dependent receptors are outer membrane transport proteins found in gram-negative bacteria, which facilitate the active transport of siderophores, enabling the uptake of essential nutrients such as iron and vitamin B12 (65). TonB-dependent receptors that have been identified in *P. aeruginosa* include FpvA (pyoverdin-iron complex receptor), FptA (pyochelin-iron complex receptor), PfeA (enterobactin-iron complex receptor), and BtuB (vitamin B12 receptor)(66, 67). The tandem duplication that we identified in the Ned 5 BIM contains the *fepA* gene, encoding for the TonB-dependent receptor, FepA. TonB-dependent receptors have been identified as bacteriophage receptors in other gram-negative bacteria, such as *E. coli* and *Salmonella enterica* (68–70). In *E. coli* under attack by phages T1, colicin M, and ϕ80, the ability of TonB-dependent receptor FhuA to act as a phage receptor appears to be dependent on the interaction of the periplasmic protein, TonB, with the receptor FhuA (69). Killmann et al. also demonstrated that FhuA mutants had decreased sensitivity to the aforementioned phages (69).

Gene duplication in bacterial genomes has been noted as a response to antimicrobial exposure, as well as being a source of functional diversity (71). A duplicated gene often results in increased gene dosage (71). We postulate that the duplicated TonB-dependent receptor in the Ned 5 BIM may be a non-functional or “decoy” phage receptor, allowing phage adsorption but not translocation of the phage genetic material into the bacteria. A similar mechanism of phage resistance has previously been described, involving the adsorption of phage to non-retractile type IV pili on *P. aeruginosa* strain K (72). Further studies would be required to test our current decoy receptor hypothesis.

We also identified prophage deletion and induction in BIMs that were not present in the vehicle control treated isolates, in two strains, PAO1 and Ned 5, respectively. Prophages occur when a temperate bacteriophage integrates its genome into the host bacterial genome, entering a lysogenic cycle, rather than causing lysis of the host (73). A vast diversity of prophages exists among *P. aeruginosa* strains, and a myriad of survival advantages conferred by prophages have been previously described (74). *P. aeruginosa* prophages have been found to resist superinfection of their host by other phages, via mechanisms including phage repressor proteins and modification of cell-surface phage receptor molecules (75, 76). The induced prophage in the Ned 5 BIM contains the gene encoding for BstA, an abortive infection system phage defense protein. BstA mediates phage resistance by interfering with phage DNA replication in the infected host bacterium (64). Induction of this prophage in the Ned 5 BIM bacterial population in response to infection by phages in CT-PA, with resulting abortive infection and prevention of CT-PA phage replication, may mediate population-level resistance to the phage cocktail.

The significance of the prophage deletion in the PAO1 BIM is unclear. Multiple mechanisms can lead to prophage induction and therefore excision of the prophage from the host bacterial genome. Events that can trigger this process include extrinsic factors that damage host bacterial DNA or interfere with DNA replication, such as ultraviolet radiation, reactive oxygen species, and antibiotics (eg., mitomycin C and fluoroquinolones) (77). The molecular mechanisms of prophage induction include an SOS response when RecA protein binds to single-stranded DNA, or expression of an antirepressor protein that binds to the phage repressor under control of LexA, resulting in induction of the lytic growth cycle (77). Stochastic fluctuations in gene expression could also result in spontaneous prophage induction, which has been observed in multiple bacterial species (77). The apparent prophage deletion in the PAO1 BIM isolates, as opposed to the presence of the prophage in the PAO1 vehicle-control treated isolate, suggests excision of the prophage from the bacterial genome has occurred in the majority of the PAO1 BIM bacterial population, without subsequent bacterial lysis. This could be explained by the deleted prophage being a cryptic prophage, a prophage that has lost the ability to lyse bacteria and form plaques but may still be induced and therefore excised from the host genome (61, 78–80). Although prophages may confer survival benefits to bacteria, they can also create a selective survival disadvantage, as has been demonstrated in nasopharyngeal *Staphylococcus aureus* (77). There are also theoretical selective advantages of prophage deletion from bacterial genomes, including decreasing the metabolic burden of extra DNA synthesis and the removal of selfish DNA elements (78).

Missense mutations in *rfaB*, a non-essential lipopolysaccharide (LPS) synthesis gene, were also identified in both PAO1 and Ned 5 BIMs. *rfaB* mutations have been shown to alter the LPS phenotype and confer resistance to Mu phage in *E. coli*. Mutations in *tagH*, also involved in LPS synthesis, as well as *pdxA*, which may be involved in LPS transport or synthesis, were also identified in the Ned 5 BIM (81–83). Multiple previous studies have shown that several *P. aeruginosa* phages use LPS as a receptor and mutations in LPS synthesis genes can confer phage resistance (84, 85). It is plausible that one of the phages in the phage cocktail used in the current study uses LPS as a receptor and that the mutations in *rfaB* confer resistance by altering the LPS core.

Chan et al’s work suggests that the development of BIMs could be exploited for therapeutic intervention by the application of phages that use receptors related to antibiotic resistance, in order to exert negative selection pressure against antibiotic-resistant traits (25). BaeS, which we found to be mutated in the Ned 5 BIM but not in the vehicle-treated control isolate, is one component of the two-component system BaeSR. BaeS is a sensor histidine kinase that detects environmental changes, leading to phosphorylation of BaeR, which then modulates downstream gene expression (86). BaeSR has been shown to influence *A. baumannii* tigecycline and cefiderocol susceptibility, as well as *E. coli* novobiocin and deoxycholate sensitivity (87–89). We also identified a mutation in *opmE*, the gene encoding the OpmE component of the *P. aeruginosa* multidrug efflux pump MexPQ-OpmE, in the Ned 5 BIM that was not present in the vehicle-treated control isolates. Mima et al. demonstrated that the introduction of *mexPQ-opmE* into an antibiotic-hypersensitive strain of *P. aeruginosa* resulted in elevated minimum inhibitory concentration values for several antibiotics, including erythromycin, norfloxacin, ciprofloxacin, and chloramphenicol (90). Our antibiotic minimum inhibitory concentration testing demonstrated increased sensitivity to chloramphenicol and ciprofloxacin in the phage-treated Ned 5 BIM, compared with both the Ned 5 parent and vehicle-treated control isolates. This would be consistent with the *opmE* frameshift mutation leading to the production of dysfunctional OpmE protein.

The N-acetylmuramoyl-L-alanine amidase (*amiB*) missense mutation identified in the USA 2 BIM is within a gene encoding for a lytic enzyme involved in peptidoglycan metabolism (91). Depletion of AmiB in *P. aeruginosa* PAO1 has previously been shown to result in increased sensitivity to gentamicin, tobramycin, vancomycin, meropenem, and irtapenem (92). Unaltered sensitivity to imipenem was observed in the same study. Yakhnina et al. postulate that the increased antibiotic sensitivity is due to increased outer membrane permeability. We observed increased meropenem sensitivity in the USA 2 BIM compared with the USA 2 vehicle-treated control on MIC assays, although the meropenem MIC of the BIM was the same as the USA 2 parent strain. Yakhnina et al. also found that AmiB is essential for normal cell growth and division in *P. aeruginosa*, although ongoing viability in an AmiB-depleted strain was possible under specific environmental conditions or with concurrent mutations in other loci. Interestingly, N-acetylmuramoyl-L-alanine amidases have also been previously identified as bacteriophage lytic enzymes (or endolysins), produced by *E. coli*, *Streptococcus mitis*, and *Bacillus subtilis* phages (93–95). Further studies are required to determine the functional significance of the *amiB* mutation that has been identified in the present study and whether it plays a role in the increased meropenem sensitivity and phage resistance that we observed in the USA 2 BIM.

Although no mutations in known antimicrobial resistance genes were identified in the PAO1 BIM compared with the PAO1 vehicle-treated control isolate, we found increased tetracycline and chloramphenicol sensitivity in the PAO1 BIM. There were also changes in carbapenem susceptibility between the PAO1 parent, control, and BIM isolates. These changes in antibiotic susceptibility may be due to changes in gene expression, which would not have been detected by whole genome sequencing.

Some of the mutations we have identified could potentially result in decreased production of bacterial virulence factors or increased susceptibility to other antimicrobials, as has been shown previously for TonB-dependent receptor BtuB mutations in *E. coli* and LPS mutations in *P. aeruginosa* (85, 96). RtcB controls the expression of virulence factors in *P. aeruginosa* and was found to be mutated in the Ned 5 BIM (97). The *fimV* gene appears to be responsible for twitching motility, a virulence factor, and was also found to be mutated in the Ned 5 BIM (98). Further studies are required to determine the functional impact of the mutations that we have identified in the current study on the isolate virulence factors.

The limitations of our study include the inability to isolate the effects of exposure of the bacterial strains to specific individual phages, as a cocktail containing four phages was used. Our study attempts to more closely replicate real-world phage therapy, where cocktails containing multiple phages are used to increase the range of sensitivity (host range) and theoretically reduce the incidence of resistance. We have identified a large number of mutations in BIMs after a biofilm treatment period of 7 days. The appearance of BIMs in bacterial cultures exposed to infecting bacteriophages has been described in numerous *in vitro* studies (31, 99, 100). Betts et al. have provided evidence of the development of phage resistance in *P. aeruginosa* through coevolution (101). Additionally, Pal et al. have shown that the rate of spontaneous mutations is increased in bacteria that have co-evolved with lytic phages (102). These mutations may affect the expression of bacterial cell surface molecules that are used by phages as receptors, resulting in phage resistance (103).

The clinical significance of the acquisition of phage resistance by bacterial isolates or the selection of such variants during phage therapy is unclear, as the majority of clinical studies of anti-*P*. *aeruginosa* phage therapy published does not report the emergence of BIMs as an outcome measure. A decrease in the proportion of *E. coli* colonies susceptible to T4 phage was described by Bruttin et al. after healthy adults ingested the phage in drinking water for two 2-day courses over 3 weeks (104). Aslam et al. describe the detection of an isolate with resistance to a *P. aeruginosa* phage cocktail 7 days after cessation of a 2-week course of intravenous and nebulized phage treatment for *P. aeruginosa* pneumonia in a bilateral lung transplant patient (105). This patient subsequently received a treatment course of the same phage cocktail with the addition of one new phage targeting the resistant strain. All subsequent isolates collected showed sensitivity to the phage cocktail, and no resistant strains were isolated during the treatment.

In conclusion, we have identified genomic changes in bacteriophage-resistant mutants of clinical *P. aeruginosa* isolates, following repeated exposure of biofilms to a phage cocktail. Mutations in genes encoding for likely phage receptors, such as TonB-dependent receptors and lipopolysaccharide, were identified in two strains. We also identified structural mutations involving prophages in two strains, which we postulate may be a bacterial host defense response against invading lytic phages. We also found mutations in several loci that are associated with virulence factors and antibiotic resistance. There were changes in antibiotic sensitivity in several of the phage-treated isolates, which were consistent with the genetic mutations identified. We are hopeful that the genomic changes in *P. aeruginosa* mutants with resistance to bacteriophages could be harnessed to inform the future application of bacteriophage therapy and to improve the efficacy of currently available antimicrobial therapies.

## Data Availability

Whole genome sequences, as well as the raw short and long reads, for the isolates in this study have been submitted to the NCBI Sequence Read Archive under BioProject number PRJNA962291.

## SUPPLEMENTAL MATERIAL

Supplemental tables spectrum.02149-24-s0001.docx Tables S1 and S2.

ASM does not own the copyrights to Supplemental Material that may be linked to, or accessed through, an article. The authors have granted ASM a non-exclusive, world-wide license to publish the Supplemental Material files. Please contact the corresponding author directly for reuse.
