# Supplementary material for: Genomic variation in Pseudomonas aeruginosa clinical respiratory isolates with de novo resistance to a bacteriophage cocktail
Source: Microbiol Spectr. 2025 Mar 31;13(5):e02149-24. doi: 10.1128/spectrum.02149-24 (PMC12054119; doi:10.1128/spectrum.02149-24)
Supplement: Supplemental tables — Tables S1 and S2. [file spectrum.02149-24-s0001.docx]

**SUPPLEMENTARY FIGURES & TABLES**

Supplementary Table 1: Table showing the Pharokka annotations of all CDS found in the PA01 deletion, visualised in Figure 1B. All CDS with an identified PHROG (ie. if there is a value in the ‘phrog’) column are likely phage genes.

| Gene | Start | Stop | Frame | PHROG | alnScore | seqIdentity | eVal | Top Hit PHROG Protein | Method | Annotation | Category |
| --- | --- | --- | --- | --- | --- | --- | --- | --- | --- | --- | --- |
| deletion_CDS_0001 | 84 | 227 | + | No_PHROG | No_PHROG | No_PHROG | No_PHROG | No_PHROG | PHANOTATE | hypothetical protein | unknown function |
| deletion_CDS_0002 | 239 | 391 | + | No_PHROG | No_PHROG | No_PHROG | No_PHROG | No_PHROG | PHANOTATE | hypothetical protein | unknown function |
| deletion_CDS_0003 | 572 | 1087 | + | 8717 | 421 | 1 | 2.40E-138 | p70508 VI_02625 | PHANOTATE | hypothetical protein | unknown function |
| deletion_CDS_0004 | 1173 | 1373 | + | 38460 | 42 | 0.75 | 6.52E-10 | MF490237_p14 | PHANOTATE | hypothetical protein | unknown function |
| deletion_CDS_0005 | 1383 | 2072 | + | 1599 | 270 | 0.617 | 4.21E-85 | p28037 VI_00883 | PHANOTATE | hypothetical protein | unknown function |
| deletion_CDS_0006 | 2383 | 2054 | - | 3944 | 114 | 0.936 | 2.07E-34 | NC_030929_p45 | PHANOTATE | hypothetical protein | unknown function |
| deletion_CDS_0007 | 3193 | 2390 | - | 56 | 194 | 0.438 | 7.11E-58 | NC_029057_p67 | PHANOTATE | DNA methyltransferase | other |
| deletion_CDS_0008 | 3598 | 3338 | - | 3580 | 77 | 0.627 | 3.02E-21 | p349592 VI_06391 | PHANOTATE | hypothetical protein | unknown function |
| deletion_CDS_0009 | 3963 | 3595 | - | 3774 | 214 | 0.846 | 8.26E-68 | p34546 VI_03090 | PHANOTATE | hypothetical protein | unknown function |
| deletion_CDS_0010 | 4589 | 3960 | - | 181 | 220 | 0.549 | 2.03E-68 | p199731 VI_00081 | PHANOTATE | endolysin | lysis |
| deletion_CDS_0011 | 4720 | 4619 | - | No_PHROG | No_PHROG | No_PHROG | No_PHROG | No_PHROG | PHANOTATE | hypothetical protein | unknown function |
| deletion_CDS_0012 | 4815 | 4726 | - | No_PHROG | No_PHROG | No_PHROG | No_PHROG | No_PHROG | PHANOTATE | hypothetical protein | unknown function |
| deletion_CDS_0013 | 5071 | 4835 | - | 5076 | 109 | 0.674 | 5.26E-32 | p96253 VI_08395 | PHANOTATE | hypothetical protein | unknown function |
| deletion_CDS_0014 | 6831 | 5071 | - | 6079 | 676 | 0.621 | 2.88E-219 | p75806 VI_12017 | PHANOTATE | tail protein | tail |
| deletion_CDS_0015 | 7688 | 6828 | - | 26277 | 361 | 0.731 | 3.33E-116 | p75807 VI_12017 | PHANOTATE | hypothetical protein | unknown function |
| deletion_CDS_0016 | 8692 | 7685 | - | 19312 | 41 | 0.96 | 1.40E-06 | p212586 VI_07536 | PHANOTATE | hypothetical protein | unknown function |
| deletion_CDS_0017 | 12534 | 8692 | - | 6128 | 1517 | 0.654 | 0 | p400704 VI_05701 | PHANOTATE | hypothetical protein | unknown function |
| deletion_CDS_0018 | 16018 | 12698 | - | 31957 | 797 | 0.457 | 8.26E-254 | p290912 VI_11476 | PHANOTATE | hypothetical protein | unknown function |
| deletion_CDS_0019 | 16793 | 16041 | - | 807 | 143 | 0.352 | 1.30E-40 | MF476925_p24 | PHANOTATE | major tail protein with Ig-like domain | tail |
| deletion_CDS_0020 | 17021 | 16821 | - | No_PHROG | No_PHROG | No_PHROG | No_PHROG | No_PHROG | PHANOTATE | hypothetical protein | unknown function |
| deletion_CDS_0021 | 17509 | 17072 | - | 22188 | 291 | 0.947 | 6.13E-94 | p361226 VI_11999 | PHANOTATE | hypothetical protein | unknown function |
| deletion_CDS_0022 | 17787 | 17506 | - | 7243 | 134 | 0.699 | 2.40E-40 | p369420 VI_11929 | PHANOTATE | hypothetical protein | unknown function |
| deletion_CDS_0023 | 18938 | 17820 | - | 29 | 89 | 0.228 | 1.05E-21 | p33806 VI_03880 | PHANOTATE | major head protein | head and packaging |
| deletion_CDS_0024 | 19521 | 18886 | - | No_PHROG | No_PHROG | No_PHROG | No_PHROG | No_PHROG | PHANOTATE | hypothetical protein | unknown function |
| deletion_CDS_0025 | 20690 | 19518 | - | 3348 | 572 | 0.762 | 8.84E-187 | p318280 VI_06662 | PHANOTATE | hypothetical protein | unknown function |
| deletion_CDS_0026 | 22140 | 20674 | - | 21 | 394 | 0.459 | 8.59E-123 | p173499 VI_06341 | PHANOTATE | portal protein | head and packaging |
| deletion_CDS_0027 | 22364 | 22140 | - | 118 | 49 | 0.401 | 2.24E-11 | p69560 VI_02299 | PHANOTATE | head-tail adaptor | connector |
| deletion_CDS_0028 | 24391 | 22376 | - | 15 | 526 | 0.456 | 4.51E-166 | p173497 VI_06341 | PHANOTATE | terminase large subunit | head and packaging |
| deletion_CDS_0029 | 24976 | 24395 | - | 57 | 59 | 0.235 | 2.29E-13 | p277333 VI_02593 | PHANOTATE | terminase small subunit | head and packaging |
| deletion_CDS_0030 | 25236 | 25087 | - | No_PHROG | No_PHROG | No_PHROG | No_PHROG | No_PHROG | PHANOTATE | hypothetical protein | unknown function |
| deletion_CDS_0031 | 25978 | 25241 | - | 1924 | 175 | 0.407 | 5.34E-52 | p318309 VI_04274 | PHANOTATE | minor tail protein | tail |
| deletion_CDS_0032 | 26271 | 25981 | - | 217 | 89 | 0.529 | 8.86E-25 | p193117 VI_11892 | PHANOTATE | holin | lysis |
| deletion_CDS_0033 | 26629 | 26264 | - | 198 | 86 | 0.434 | 1.86E-23 | p387102 VI_09157 | PHANOTATE | holin | lysis |
| deletion_CDS_0034 | 26737 | 26642 | - | No_PHROG | No_PHROG | No_PHROG | No_PHROG | No_PHROG | PHANOTATE | hypothetical protein | unknown function |
| deletion_CDS_0035 | 27385 | 26828 | - | 4300 | 67 | 0.326 | 7.67E-17 | p71594 VI_07287 | PHANOTATE | hypothetical protein | unknown function |
| deletion_CDS_0036 | 27725 | 27378 | - | No_PHROG | No_PHROG | No_PHROG | No_PHROG | No_PHROG | PHANOTATE | hypothetical protein | unknown function |
| deletion_CDS_0037 | 30451 | 27722 | - | 4762 | 243 | 0.375 | 9.80E-69 | p277771 VI_05561 | PHANOTATE | hypothetical protein | unknown function |
| deletion_CDS_0038 | 30704 | 30486 | - | 14685 | 56 | 0.401 | 2.40E-13 | p222120 VI_08569 | PHANOTATE | hypothetical protein | unknown function |
| deletion_CDS_0039 | 30688 | 30903 | + | No_PHROG | No_PHROG | No_PHROG | No_PHROG | No_PHROG | PHANOTATE | hypothetical protein | unknown function |
| deletion_CDS_0040 | 30996 | 31580 | + | No_PHROG | No_PHROG | No_PHROG | No_PHROG | No_PHROG | PHANOTATE | hypothetical protein | unknown function |
| deletion_CDS_0041 | 31890 | 31654 | - | No_PHROG | No_PHROG | No_PHROG | No_PHROG | No_PHROG | PHANOTATE | hypothetical protein | unknown function |
| deletion_CDS_0042 | 31969 | 32634 | + | 4 | 54 | 0.233 | 3.45E-11 | p259758 VI_06270 | PHANOTATE | transcriptional repressor | transcription regulation |
| deletion_CDS_0043 | 32645 | 32743 | + | No_PHROG | No_PHROG | No_PHROG | No_PHROG | No_PHROG | PHANOTATE | hypothetical protein | unknown function |
| deletion_CDS_0044 | 32798 | 33007 | + | 13491 | 57 | 0.437 | 2.86E-14 | p186771 VI_09509 | PHANOTATE | hypothetical protein | unknown function |
| deletion_CDS_0045 | 32973 | 33875 | + | 139 | 111 | 0.362 | 1.44E-30 | KX507046_p83 | PHANOTATE | deoxynucleoside monophosphate kinase | other |
| deletion_CDS_0046 | 33887 | 34171 | + | 5689 | 98 | 0.583 | 3.36E-28 | p318296 VI_06662 | PHANOTATE | hypothetical protein | unknown function |
| deletion_CDS_0047 | 34168 | 34536 | + | 8889 | 126 | 0.572 | 4.97E-37 | p318297 VI_06662 | PHANOTATE | hypothetical protein | unknown function |
| deletion_CDS_0048 | 34533 | 34769 | + | 1300 | 52 | 0.437 | 2.73E-12 | p258877 VI_10210 | PHANOTATE | excisionase | integration and excision |
| deletion_CDS_0049 | 34881 | 34991 | + | No_PHROG | No_PHROG | No_PHROG | No_PHROG | No_PHROG | PHANOTATE | hypothetical protein | unknown function |
| deletion_CDS_0050 | 34988 | 35326 | + | 204 | 79 | 0.4 | 3.83E-21 | p371814 VI_11565 | PHANOTATE | hypothetical protein | unknown function |
| deletion_CDS_0051 | 37005 | 35428 | - | No_PHROG | No_PHROG | No_PHROG | No_PHROG | No_PHROG | PHANOTATE | hypothetical protein | unknown function |
| deletion_CDS_0052 | 36999 | 37544 | + | 318 | 225 | 0.642 | 2.20E-70 | p402927 VI_10515 | PHANOTATE | deoxyribonucleoside 5' monophosphate phosphatase | other |
| deletion_CDS_0053 | 37541 | 39694 | + | 23072 | 289 | 0.44 | 7.87E-89 | p191613 VI_09061 | PHANOTATE | DNA methyltransferase | other |
| deletion_CDS_0054 | 39646 | 40122 | + | 3190 | 258 | 0.868 | 6.55E-82 | NC_027986_p8 | PHANOTATE | hypothetical protein | unknown function |
| deletion_CDS_0055 | 40109 | 40855 | + | 11884 | 53 | 0.413 | 2.63E-10 | p128079 VI_03160 | PHANOTATE | hypothetical protein | unknown function |
| deletion_CDS_0056 | 40855 | 41169 | + | No_PHROG | No_PHROG | No_PHROG | No_PHROG | No_PHROG | PHANOTATE | hypothetical protein | unknown function |
| deletion_CDS_0057 | 41162 | 41281 | + | 3765 | 47 | 0.592 | 3.39E-11 | p128080 VI_03160 | PHANOTATE | hypothetical protein | unknown function |
| deletion_CDS_0058 | 41281 | 41487 | + | 2790 | 55 | 0.479 | 4.73E-13 | p43040 VI_12007 | PHANOTATE | excisionase and transcriptional regulator | integration and excision |
| deletion_CDS_0059 | 42695 | 41493 | - | 3423 | 59 | 0.429 | 6.01E-13 | p166161 VI_03799 | PHANOTATE | hypothetical protein | unknown function |
| deletion_CDS_0060 | 42847 | 42728 | - | No_PHROG | No_PHROG | No_PHROG | No_PHROG | No_PHROG | PHANOTATE | hypothetical protein | unknown function |

Supplementary Table 2: Table showing all small nucleotide variants found in the Ned 5 BIM compared to the vehicle-treated control according to Snippy. For variant type, ‘INS’ refers to insertion, ‘DEL’ refers to deletion, and ‘SNP’ refers to single nucleotide polymorphism. ‘REF’ refers to the sequence in the untreated control, ‘ALT’ refers to the sequence in the BIM. Evidence refers to the depth of coverage of both the REF and ALT allelles.

| **Position** | **Variant Type** | **REF** | **ALT** | **Evidence** | **Coding sequence?** | **Effect** | **Gene** | **Product** | **Uniref90 Accession (Bakta)** |
| --- | --- | --- | --- | --- | --- | --- | --- | --- | --- |
| 13024 | ins | G | GC | GC:33 G:0 |  |  |  |  |  |
| 96662 | ins | C | CG | CG:34 C:0 | CDS | frameshift_variant c.608dupC p.Arg204fs |  | type VI secretion system-associated FHA domain protein TagH | UPI00053D4030 |
| 134742 | ins | T | TA | TA:67 T:0 |  |  |  |  |  |
| 336483 | ins | C | CG | CG:92 C:1 | CDS | frameshift_variant c.173dupC p.Leu59fs |  | hypothetical protein | None |
| 357399 | ins | G | GA | GA:75 G:0 |  |  |  |  |  |
| 572001 | ins | C | CT | CT:58 C:0 |  |  |  |  |  |
| 572039 | ins | T | TG | TG:41 T:1 |  |  |  |  |  |
| 658908 | ins | A | AGGG | AGGG:52 A:0 | CDS | conservative_inframe_insertion c.89_91dupCCC p.Pro30dup | pdxA | 4-hydroxythreonine-4-phosphate dehydrogenase PdxA | Q9I5U4 |
| 858161 | ins | T | TC | TC:77 T:0 |  |  |  |  |  |
| 858169 | ins | C | CGG | CGG:74 C:0 |  |  |  |  |  |
| 1024321 | ins | C | CT | CT:60 C:0 |  |  |  |  |  |
| 1165941 | ins | G | GA | GA:36 G:0 |  |  |  |  |  |
| 1205142 | ins | C | CG | CG:74 C:0 |  |  |  |  |  |
| 1507850 | snp | T | C | C:47 T:0 |  |  |  |  |  |
| 1507857 | snp | A | G | G:50 A:0 |  |  |  |  |  |
| 1516436 | ins | G | GT | GT:67 G:0 |  |  |  |  |  |
| 1589424 | ins | A | AG | AG:42 A:0 |  |  |  |  |  |
| 1663450 | ins | G | GT | GT:48 G:0 |  |  |  |  |  |
| 1809496 | ins | G | GCC | GCC:48 G:0 |  |  |  |  |  |
| 1815592 | snp | A | G | G:64 A:0 | CDS | missense_variant c.1463A>G p.Gln488Arg | czcO | FAD-containing monooxygenase EthA | A0A080VSN6 |
| 1830880 | ins | C | CT | CT:57 C:1 | CDS | frameshift_variant c.1132dupA p.Arg378fs | mltA | transglycosylase | A0A2R3IXK2 |
| 1876277 | ins | C | CG | CG:46 C:0 |  |  |  |  |  |
| 1953221 | ins | G | GC | GC:60 G:3 |  |  |  |  |  |
| 1984866 | ins | A | AG | AG:61 A:0 |  |  |  |  |  |
| 2035180 | ins | A | AC | AC:87 A:0 | CDS | frameshift_variant c.705dupC p.Pro237fs |  | UvrD-helicase domain-containing protein | UPI00066CA611 |
| 2096480 | ins | A | AG | AG:74 A:0 | CDS | frameshift_variant c.1806dupG p.Pro603fs |  | Phosphorelay protein LuxU | Q5E3S0 (Uniref50) |
| 2151764 | ins | T | TC | TC:55 T:0 |  |  |  |  |  |
| 2162259 | ins | G | GA | GA:67 G:0 |  |  |  |  |  |
| 2162280 | del | CA | C | C:62 CA:0 |  |  |  |  |  |
| 2162305 | complex | AGC | GAGCG | GAGCG:56 AGC:0 |  |  |  |  |  |
| 2247600 | ins | G | GC | GC:68 G:0 |  |  |  |  |  |
| 2564380 | ins | C | CT | CT:57 C:0 |  |  |  |  |  |
| 3064834 | ins | C | CG | CG:44 C:1 |  |  |  |  |  |
| 3115706 | ins | G | GT | GT:45 G:0 | CDS | frameshift_variant c.4775dupA p.Asn1592fs | entF | EntF, seryl-AMP synthase component of non-ribosomal peptide synthetase | A0A643EKV7 |
| 3133701 | ins | A | AC | AC:77 A:0 | CDS | frameshift_variant c.2232dupG p.Ser745fs |  | amino acid adenylation domain-containing protein | UPI000F51FCBD |
| 3142529 | del | CGA | C | C:29 CGA:0 |  |  |  |  |  |
| 3242350 | snp | C | T | T:62 C:0 | CDS | missense_variant c.1226C>T p.Thr409Ile | baeS | two-component sensor histidine kinase | A0A069PZI5 |
| 3260473 | ins | C | CT | CT:34 C:0 |  |  |  |  |  |
| 3342481 | complex | T | GGGG | GGGG:33 T:1 |  |  |  |  |  |
| 3477161 | snp | G | A | A:73 G:0 | CDS | missense_variant c.851G>A p.Arg284His |  | RNA-splicing ligase RtcB | A0A0P0AID0 |
| 3598485 | ins | G | GCCC | GCCC:38 G:0 |  |  |  |  |  |
| 3689752 | ins | A | ACC | ACC:59 A:0 | CDS | intragenic_variant n.3689752_3689753insCC | aspB | aminotransferase | A0A073A5B3 (Uniref100) |
| 3690082 | ins | T | TC | TC:69 T:0 | CDS | frameshift_variant c.285dupC p.Gly96fs |  | hypothetical protein | None |
| 4195574 | ins | C | CT | CT:76 C:1 |  |  |  |  |  |
| 4237505 | ins | C | CTA | CTA:59 C:0 |  |  |  |  |  |
| 4255141 | ins | C | CG | CG:48 C:1 | CDS | frameshift_variant c.1204dupC p.Arg402fs |  | Tfp pilus assembly protein FimV | A0A485FKG2 |
| 4346348 | ins | T | TG | TG:27 T:1 |  |  |  |  |  |
| 4346375 | ins | T | TC | TC:29 T:0 |  |  |  |  |  |
| 4346475 | ins | T | TC | TC:20 T:0 |  |  |  |  |  |
| 4346574 | ins | T | TCC | TCC:11 T:0 |  |  |  |  |  |
| 4346674 | ins | T | TC | TC:9 T:0 |  |  |  |  |  |
| 4377477 | ins | G | GA | GA:36 G:0 |  |  |  |  |  |
| 4377591 | ins | G | GA | GA:42 G:0 |  |  |  |  |  |
| 4384071 | ins | T | TC | TC:59 T:1 |  |  |  |  |  |
| 4417443 | ins | G | GC | GC:51 G:0 | CDS | frameshift_variant c.1383dupG p.Leu462fs | tolC | Outer membrane efflux protein | A0A509JP07 |
| 4426372 | ins | G | GC | GC:19 G:0 |  |  |  |  |  |
| 4426394 | ins | T | TC | TC:22 T:0 |  |  |  |  |  |
| 4468110 | ins | A | AC | AC:51 A:0 |  |  |  |  |  |
| 4718076 | ins | G | GC | GC:58 G:0 |  |  |  |  |  |
| 4777540 | ins | T | TA | TA:62 T:0 | CDS | frameshift_variant c.967_968insA p.Val323fs |  | ABC transporter substrate-binding protein | A0A080VS54 |
| 4790748 | del | AG | A | A:55 AG:0 |  |  |  |  |  |
| 4809043 | ins | T | TC | TC:67 T:0 |  |  |  |  |  |
| 4823740 | snp | A | G | G:54 A:0 | CDS | synonymous_variant c.426T>C p.Asn142Asn | narK | MFS transporter | A0A069Q5D4 |
| 4823749 | snp | A | G | G:59 A:0 | CDS | synonymous_variant c.417T>C p.Gly139Gly | narK | MFS transporter | A0A069Q5D4 |
| 4868134 | complex | T | CTC | CTC:56 T:0 |  |  |  |  |  |
| 4917595 | ins | C | CG | CG:59 C:0 |  |  |  |  |  |
| 5038318 | ins | C | CG | CG:38 C:0 | CDS | frameshift_variant c.709dupC p.Arg237fs |  | hypothetical protein | A0A3M5ER77 |
| 5103395 | ins | T | TCC | TCC:46 T:0 |  |  |  |  |  |
| 5284826 | mnp | GG | TA | TA:53 GG:0 |  |  |  |  |  |
| 5284857 | snp | G | A | A:71 G:0 |  |  |  |  |  |
| 5284863 | del | CA | C | C:73 CA:0 |  |  |  |  |  |
| 5284873 | snp | T | A | A:79 T:0 |  |  |  |  |  |
| 5387570 | ins | A | AAG | AAG:69 A:0 |  |  |  |  |  |
| 5429016 | ins | G | GA | GA:56 G:1 |  |  |  |  |  |
| 5490482 | mnp | GA | AG | AG:77 GA:1 |  |  |  |  |  |
| 5632533 | del | ACTTGGT | A | A:69 ACTTGGT:0 | CDS | conservative_inframe_deletion c.556_561delACCAAG p.Thr186_Lys187del | rfaB | glycosyltransferase | A0A2R3IUW8 |
| 5652434 | ins | C | CA | CA:70 C:0 |  |  |  |  |  |
| 5899409 | ins | T | TCC | TCC:45 T:0 |  |  |  |  |  |
| 6042341 | ins | T | TC | TC:46 T:0 |  |  |  |  |  |
| 6092810 | ins | G | GC | GC:49 G:0 |  |  |  |  |  |
| 6112384 | snp | C | G | G:73 C:0 | CDS | missense_variant c.533C>G p.Ala178Gly | fadH2 | sarcosine oxidase subunit alpha | UPI0003B9BA99 (Uniref100) |
| 6119601 | ins | G | GC | GC:41 G:0 |  |  |  |  |  |
